# Serotonin reuptake inhibitors and bleeding risks related to elderly submitted to major orthopedic surgery

**DOI:** 10.31744/einstein_journal/2019ED5214

**Published:** 2019-10-03

**Authors:** Felicio Savioli

**Affiliations:** 1 Hospital Alemão Oswaldo Cruz São PauloSP Brazil Hospital Alemão Oswaldo Cruz, São Paulo, SP, Brazil.

Depression is a major global public health problem, the leading cause of disability with an estimated global prevalence of 4.7%, and the eleventh leading cause of global disease burden.^1^ Among elderly individuals, depression is one of the most prevalent mental disorder, a common cause of disability, and reduced life-satisfaction. Along with the worldwide increase in number of older adults, a better understanding of depression among older people is highly valuable from clinical and public health perspectives.^2^ Late-life depression is a common psychiatric disorder that decrease older people’s quality of life. Around 14% of individuals older than 55 years have depression including 2% with major depression. Factors associated with late-life depression includes female sex, chronic somatic illness, cognitive impairment, functional impairment, lack of close social contacts, personality traits, stressful life-events, and a history of depression.^3^

The use of antidepressants by elderly patients has some risks. However, untreated or inadequately treated depression is more dangerous and can lead to other adverse health outcomes such as malnutrition, poor hydration, weakness from a lack of physical activity, functional decline, decrease in quality of life, and ultimately, suicide and death.

Selective serotonin reuptake inhibitors (SSRI) are well established as first line treatment for old-age depression. However, different studies showed that SSRIs (paroxetine, fluoxetine, fluvoxamine, sertraline, and venlafaxine) increases the risk of abnormal bleeding, including major surgery.

Bleeding complications of surgery constitute a substantial source of financial burden, patient morbidity, and mortality. Significant intraoperative bleeding may require blood transfusion and all the inherent risks. Even with meticulous surgical technique, certain operative procedures have a well-documented associated blood loss. Major orthopedic operations, such as total hip or knee replacement, result in higher volumes of blood loss.

Platelets take up and store in dense granules the serotonin synthesized by enterochromaffin cells in the gut via the serotonin transporter located in the membrane. Serotonin is essential to normal platelet function. A critical component of platelet activation is serotonin secretion, which has a number of different effects, including strong vasoactive properties through direct action on serotonin receptors and nitric oxide production, the potentiation of the aggregation induced by adenosine diphosphate, epinephrine, and collagen and the enhancement of fibrin formation.^4^

Serotonin is considered by itself a relatively weak platelet activator, but it has great potentiation for aggregation induced by adenosine diphosphate, epinephrine and collagen. Additionally, recent perfusion studies have shown that serotonin enhances fibrin formation and increases the surface covered by large platelet aggregation, therefore, actually inducing a thrombogenic state in circulating human blood.^5^ Different randomized controlled trial showed that SSRI changes laboratory tests during assessment of function of primary hemostasis and clotting cascade.^6 - 8^ For these reasons, SSRI are described as drugs potentially associated with enhanced bleeding risks.

The epidemiological evidence broadly supports a moderately increased risk of gastrointestinal bleeding among elderly patients associated with the use of SSRI, but this depends on patient susceptibility and the presence of other risk factors, such as ageing, previous history of upper gastrointestinal bleeding or peptic ulcer, and use of nonsteroidal anti-inflammatory drugs, oral anticoagulants, antiplatelet drugs or corticosteroids. However, few studies have analyzed the risk of bleeding while under SSRI for patients who underwent major orthopedic surgery.

Major orthopedic surgery is associated with higher risk of postoperative complications compared with other types of procedures. Furthermore, patients who are candidates for major orthopedic surgery are often older than 65-year-old. With aging, the onset of comorbidities and the impairment of hepatic, renal and cardiac function increase not only the risk of developing venous thromboembolism, but also the incidence of major bleeding.

Total hip arthroplasty (THA) and total knee arthroplasty (TKA) have been performed with an increasing frequency, with estimative of more than 300,000 procedures annually only for THA.^9^

In a retrospective cohort study, Schutte et al., showed that SSRI users undergoing hip surgery had an increased risk for blood transfusion during admission, and concluded that this antidepressant drugs should be considered a risk indicator for increased blood loss among patients admitted for hip surgery.^10^

Movig et al., in a retrospective study including 520 patients who underwent major orthopedic surgery, observed that the risk of blood transfusion increased four times in group receiving SSRI compared with non-users. In addition, they concluded that bleeding and blood transfusion were associated with this antidepressant drugs.^11^ However, van Haelst et al., conducted a retrospective cohort study with patients who underwent THA and did not find association with perioperative transfusion requirements regarding patients receiving SSRI treatment.^12^

Depression is one of the most common mental disorders among old adults and serotonin reuptake inhibitor are commonly prescribed for this population. Furthermore, major orthopedic surgeries are frequently performed in elderly patients and they are associated with blood loss. Once SSRI impairs hemostasis, it is reasonable that this antidepressant drugs may increase blood transfusion in the post-operative of major orthopedic surgery. A validated prospective study among elderly population should be conducted to investigate this hypothesis.
