# Supplementary material for: Serotonin reuptake inhibitors and bleeding risks related to elderly submitted to major orthopedic surgery
Source: Einstein (Sao Paulo). 2019 Oct 3;17(4):eED5214. doi: 10.31744/einstein_journal/2019ED5214 (PMC6777648; doi:10.31744/einstein_journal/2019ED5214)
Supplement: Translation

# Translation: Inibidores da recaptação de serotonina associados ao risco de sangramento em idosos submetidos à cirurgia ortopédica

**Authors:** Felicio Savioli

**Affiliations:** 1 Brasil Hospital Alemão Oswaldo Cruz, São Paulo, SP, Brasil.

A depressão é um dos principais problemas de saúde pública global, e a principal causa de incapacidade, com estimativa global de 4,7%, constituindo a 11º principal causa global de carga de doença.^1^ Entre idosos, a depressão é uma das doenças mentais mais prevalentes, que comumente pode levar à incapacidade e à redução da satisfação da qualidade de vida. Juntamente do aumento da população de idosos, a melhor compreensão da depressão é altamente valiosa, a partir de perspectivas clínicas e públicas.^2^

A depressão tardia é um distúrbio psiquiátrico comum, que reduz a qualidade de vida entre idosos. Cerca de 14% dos indivíduos com mais de 55 anos têm depressão, incluindo 2% com transtorno depressivo grave. Os fatores associados com a depressão tardia incluem sexo feminino, doença somática crônica, lesão cognitiva, lesão funcional, falta de contato social, traços de personalidade, eventos de vida estressantes e histórico de depressão.^3^

O uso de antidepressivos por pacientes idosos tem alguns riscos. Porém, a depressão não tratada ou inadequada é muito mais perigosa e pode levar a outros resultados adversos de saúde, como desnutrição, desidratação, fraqueza por falta de atividade física, declínio funcional, redução na qualidade de vida e, por último, suicídio e morte.

Os inibidores seletivos da recaptação da serotonina (ISRS) são bem estabelecidos como tratamento de primeira linha para depressão em idosos. Porém, diferentes estudos mostram que os ISRSs (paroxetina, fluoxetina, fluvoxamina, sertralina e venlafaxina) aumentam o risco de sangramento, inclusive em grandes cirurgias.

Complicações cirúrgicas relacionadas a sangramentos são causas de morbidade e mortalidade em pacientes idosos. O sangramento intraoperatório pode requerer transfusão sanguínea, além de oferecer os riscos inerentes à transfusão de hemoderivados. Mesmo com técnicas cirúrgicas aperfeiçoadas, alguns procedimentos cirúrgicos estão associados à perda sanguínea. As grandes cirurgias ortopédicas, como artroplastia de joelho de quadril, resultam em perda de grandes quantidade de sangue.

As plaquetas armazenam em densos grânulos a serotonina sintetizada pelas células enterocromáticas no estômago, por meio do transportador de serotonina localizado em sua membrana. A serotonina é essencial para o funcionamento normal da plaqueta. Um componente fundamental para a ativação da plaqueta é a secreção de serotonina, que possui diferentes funções, incluindo fortes propriedades vasoativas, por meio de ação direta nos receptores de serotonina e produção de óxido nítrico; potencial de agregação induzido por difosfato de adenosina, epinefrina e colágeno; e melhora de formação fibrina.^4^

A serotonina é considerada um ativador de plaqueta relativamente fraco, mas tem grande potencial agregador induzido pelo difosfato de adenosina, pela epinefrina e pelo colágeno. Além disso, estudos recentes de perfusão têm mostrado que a serotonina melhora a formação de fibrina e aumenta a superfície de agregação da plaquetas, mas a serotonina promove o estágio trombogênico na circulação sanguínea humana.^5^

Diferentes estudos randomizados controlados mostraram que os ISRS promovem alteração na hemostasia primária e na cascata de coagulação.^6 - 8^ Por esses motivos, os ISRS são descritos como medicamentos potencialmente associados ao risco de sangramento.

A evidência epidemiológica sugere o aumento de risco moderado de sangramento gastrintestinal entre pacientes idosos associado com uso de ISRS. Contudo, isso depende da suscetibilidade do paciente e da presença de outros fatores de risco, como idade, histórico de sangramento gastrointestinal superior ou úlcera péptica, e uso de medicamentos anti-inflamatórios não esteroidais, anticoagulantes orais, medicamentos antiplaquetários ou corticosteroides. Porém, poucos estudos analisaram o risco de sangramento associado a ISRS em pacientes submetidos à cirurgia ortopédica de grande porte.

A cirurgia ortopédica de grande porte está associada ao alto risco de complicações pós-operatórias comparada com outros tipos de procedimentos. Além disso, os pacientes candidatos à cirurgia ortopédica maior possuem, geralmente, mais que 65 anos. Com o aumento da idade, o aparecimento de comorbidades e o comprometimento das funções hepática, renal e cardíaca aumentam o risco de desenvolvimento de tromboembolismo venoso, além do aumento da incidência de sangramento maior.

A artroplastia total de quadril (ATQ) e artroplastia total de joelho (ATJ) têm sido realizadas com relativa frequência, com estimativa de mais de 300 mil procedimentos anualmente.^9^

Em estudo de coorte retrospectivo, Schutte et al., mostraram que usuários da ISRS realizando cirurgia de quadril tinham risco aumentado para transfusão sanguínea durante a admissão, concluindo que esses medicamentos antidepressivos deveriam ser considerados fator de risco para aumento de hemorragia entre pacientes admitidos para cirurgia de quadril.^10^

Movig et al., em estudo retrospectivo, incluindo 520 pacientes que realizaram cirurgia ortopédica maior, observaram que o risco de transfusão sanguínea aumentou quatro vezes no grupo que recebeu ISRS comparado com não usuários. Além disso, conclui-se que o sangramento e a transfusão sanguínea foram associados com os medicamentos antidepressivos.^11^ Porém, van Haelst et al., conduziram estudo de coorte retrospectivo com pacientes que realizaram ATQ e não encontraram associação com necessidades de transfusão perioperatória, em relação a pacientes que receberam tratamento com ISRS.^12^

A depressão é uma dos distúrbios mentais mais comuns entre idosos, e os inibidores da recaptação de serotonina são comumente prescritos para esta população. Ainda, as cirurgias ortopédicas maiores são realizadas com frequência em pacientes idosos e estão associadas ao aumento do risco de hemorragia. Uma vez que os ISRS podem causar danos à hemostasia, é possível que estes medicamentos antidepressivos aumentem a necessidade de transfusão sanguínea em casos de cirurgias ortopédicas maiores. Um estudo prospectivo validado entre a população idosa deveria ser conduzido para investigar esta hipótese.
